# Endobronchial ultrasound-guided transbronchial needle aspiration in patients with previously treated malignancies: diagnostic performance and predictive value

**DOI:** 10.1186/s12890-022-02266-7

**Published:** 2022-12-09

**Authors:** Yan Yan, Zhilong Wang, Wanpu Yan, Shijie Li, Qi Wu

**Affiliations:** 1 Key Laboratory of Carcinogenesis and Translational Research (Ministry of Education), Department of Endoscopy, Peking University Cancer Hospital & Institute, Beijing, China; 2 Key Laboratory of Carcinogenesis and Translational Research (Ministry of Education), Department of Radiology, Peking University Cancer Hospital & Institute, Beijing, China; 3 Key Laboratory of Carcinogenesis and Translational Research (Ministry of Education), Department of Thoracic Surgery, Peking University Cancer Hospital & Institute, Beijing, China

**Keywords:** Endobronchial ultrasound-guided transbronchial needle aspiration (EBUS-TBNA), Post treatment, Ultrasonographic feature, Lymphadenopathy

## Abstract

**Background:**

Endobronchial ultrasound-guided transbronchial needle aspiration is a minimally invasive and effective sampling approach for patients with mediastinal or hilar lymphadenopathy. Increased recognition of the ultrasonographic features revealed the value of its images in predicting mediastinal lymph node malignancy. However, its diagnostic validity and the predictive value of its ultrasonographic features have not been demonstrated well in patients after systemic anti-tumor therapy. This study aimed to evaluate the efficiency of endobronchial ultrasound-guided transbronchial needle aspiration in patients with suspicious lymph nodes after anti-tumor therapy.

**Methods:**

We retrospectively reviewed cases of endobronchial ultrasound-guided transbronchial needle aspiration performed between January 2019 and August 2021 at a single tertiary hospital center. Patients with suspected mediastinal or hilar lymph nodes within 5 years of systemic anti-tumor therapy were enrolled. Final diagnoses were determined by pathologic diagnoses of samples from transbronchial needle aspiration, surgery, or follow-up for at least 6 months. Ultrasonographic features were analyzed to assess the predictive value of malignant lymph nodes after treatment.

**Results:**

Overall, 168 lymph nodes of 138 patients were analyzed. Among 110 (65.5%) malignant lymph nodes, 75 originated from lung cancers; the other 35 were from other malignancies. No complications related to endobronchial ultrasound-guided transbronchial needle aspiration were observed. Of 58 negative results of transbronchial needle aspiration, 51 were proven to be true negatives; 7 were false. The overall sensitivity and the negative predictive value were 94.02% and 87.93%, respectively. Univariate and multivariate analysis revealed the absence of central hilar structure and short axis > 10 mm as independent predictive factors for malignancy.

**Conclusions:**

Endobronchial ultrasound-guided transbronchial needle aspiration performs satisfactorily in diagnosing mediastinal and hilar lymphadenopathy even after anti-tumor treatment.

## Background

Currently, increasingly more patients with various malignancies benefit from anti-tumor therapy and have longer survival. After treatment of the primary tumor, some patients may display a suspiciously enlarged intrathoracic lymph node (LN) due to metastasis or recurrence of the primary tumor or a secondary tumor, as well as benign diseases. To decide on a treatment regimen, the cause of the lymphadenopathy needs to be identified. Especially in lung cancer, neoadjuvant therapy is introduced for better prognosis. In certain cases, immunotherapy can cause enlargement of the intrathoracic LNs, like immunological reactions [1]. If the status is misdiagnosed as progress, treatment might be interrupted or abandoned. Thus, identifying LN status and obtaining high-quality samples are increasingly gaining relevance in clinical practice.

The effectiveness and safety of endobronchial ultrasound-guided transbronchial needle aspiration (EBUS-TBNA) has been well demonstrated in various clinical settings [2–7]. Increasingly more targeted treatments and immunotherapies are available; the need for minimally invasive sampling procedures like EBUS-TBNA before and after treatment, or even during treatment, has also significantly increased [4, 5, 8–11]. However, few studies have depicted the diagnostic yield and safety of EBUS-TBNA in patients with previously treated cancer, especially those with non-lung malignancies. For this subgroup, LN status is extremely important because pathological diagnosis is pivotal for management but the majority of patients may not have the opportunity or will to undergo additional surgery only for specimen acquisition. EBUS-TBNA sampling plays a crucial role in pathological diagnosis and molecular testing. Moreover, increased recognition of ultrasonographic features revealed their predictive value for malignancy; several models or scores have been developed to guide clinicians towards better sampling and understanding of the positive or negative pathology [12–17]; however, after anti-tumor treatment, few feature characteristics are described or analyzed.

This study aimed to evaluate the use of EBUS-TBNA in patients with suspicious LNs after anti-tumor therapy for lung cancer or other malignancies.

In the present study, data of patients with enlarged mediastinal or hilar LNs who underwent EBUS-TBNA between January 2019 and August 2021, at a single tertiary hospital center within 5 years after systemic anti-tumor therapy, were retrospectively reviewed. During EBUS, ultrasonographic features, including short axis, margin, central hilar structure (CHS), round shape, and necrosis, were identified and recorded in real-time. The diagnostic value of EBUS for these LNs after treatment was investigated. In addition, the predictive value of the EBUS features was assessed for malignancy.

## Patients and methods

Patients with suspected intrathoracic LNs who had undergone any systemic anti-tumor therapy because of any malignancy within 5 years were enrolled from January 2019 to August 2021. EBUS was performed by three experienced endoscopists, mainly under surface anesthesia. Rapid on-site cytological evaluation was unavailable. EBUS features were described in medical documents during the procedure before the pathology results were revealed.

### Patient selection

The inclusion criteria were as follows: (i) previously underwent systemic anti-tumor therapy within 5 years; (ii) suspected intrathoracic LN malignancy by clinical observation (Computed tomography (CT) scanning with contrast and/or positron emission tomography (PET)). The detailed criteria was: (i) the short diameter of the lymph node ≥ 1 cm or necrosis of lymph nodes (CT scanning); (ii) the SUVmax ≥ 2.5 (PET/CT); (iii) successful EBUS-TBNA with a pathological result; (iv) if EBUS-TBNA specimen was negative, additional specimen was acquired by surgery (VATS surgery, thoracotomy or mediastinoscopic surgery), or no progress was observed in a 6-month clinical and radiological follow-up (either CT or PET/CT as clinically needed) without changing current treatment regimen.

### EBUS-TBNA procedure

All patients empty stomach (nil by mouth) for at least 4 h before undergoing EBUS-TBNA. Routinely surface anesthesia (2% lidocaine saline solution inhalation for 20 min before the procedure and timely spraying as needed during the procedure) was used. If the patient could not tolerate EBUS, general anesthesia was administered. First, the bronchoscope (BF-UC260F-OL8, Olympus, Tokyo, Japan; EB-530US, Fujifilm, Tokyo, Japan) was inserted into the trachea and bronchial tree through the nostrils or mouth. After scope insertion, lidocaine saline solution was sprayed onto the mucosa of the airway for anesthesia as needed.

After identifying the targeted LNs, ultrasound images were saved and features described. Punctures by a 22- or 25-gauge needle, approximately 1–3 times( in rare cases a maximum of 5 punctures if the specimen was not sufficient), were needed to aspirate enough samples, which were smeared on 2–4 slides and collected in a bottle that containedcytopreservation solution. The core tissue was then expelled into a bottle containing 40% neutral buffered formalin for histological examination, while the remaining aspirate and other needle passages were used for cytological examination.

All patients or their families provided written informed consent prior to any medical examination or treatment. This study was approved by the Beijing Cancer Hospital Research Ethics Committee and conducted according to the guidelines of the Declaration of Helsinki.

### Statistical analysis

#### Validity of EBUS-TBNA for previously treated LNs

Sensitivity, negative predictive value (NPV), specificity, and positive predictive value (PPV) were calculated using standard formulae. Prior to EBUS-TBNA, radiological evaluation, such as contrast CT and/or PET was conducted for the mediastinal and/or hilar LNs. Final diagnoses were carefully affirmed; positive results were determined by pathology reports of EBUS-TBNA specimens. Malignant TBNA pathology would not need confirmation by a follow-up surgery. Negative reports were confirmed using specimens from mediastinoscopy, surgery, or a at least 6-month clinical and radiological follow-up.

No EBUS-TBNA procedure-related complications such as pneumothorax, mediastinal abscess, or excessive bleeding, were observed during this study.

#### Predictive value of the ultrasonographic features in previously treated LNs

The LN was used as the unit of analysis. Quantifiable variables are presented as mean ± standard deviation, or number (percentage). Categorical variables are analyzed using Pearson’s Chi-square analysis. Several ultrasonographic features which were suggested as predictive factors (margin, CHS, small axis length, and small axis length to long axis length) are included.

Univariate binary logistic regression and multivariate logistic regression was completed (significance level *p* < 0.05).

## Results

### Patients’ characteristics

Between January 2019 and August 2021, a total of 168 LNs of 138 patients who underwent EBUS-TBNA met the inclusion criteria.

As shown in Table 1, 138 patients were finally enrolled in this study. The mean age of patients was 59.44 years (range: 23 to 77) (standard deviation, 10 years); 85 patients (61.6%) were male and 53 (38.4%) were female. Overall, 168 LN stations were sampled: stations 7 and 4 were the most common sites.

**Table 1:** Patient demographics and biopsied LNs characteristics

Patient or LN characteristics	Study sample
No. of patients	138
No. of LNs	168
Age, year	
Median	59.44
Range	23–77
Sex	
Male	85
Female	53
Radiology	
Chest CT scan	144
PET-CT	22
Frequency of biopsied LN stations	
7	46
4R	62
4L	21
10–11R	25
10–11L	12
Others	2

The final pathological diagnosis was malignancy in 110 (65.5%) LNs; 75 LNs were diagnosed with lung cancer. Other malignancies revealed in 35 patients included colon and rectal adenocarcinoma [5], esophageal cancer [3], Squamous cell carcinoma (SCC) of head and neck [5], cervical carcinoma [5], breast cancer [4], prostatic cancer [2], liver cancer [4], renal cancer [3], malignant melanoma [2], hemangiopericytoma [1], and thymic SCC [1] (Table 2). Benign causes of LN enlargement were found in 51 LNs. Pathology with granuloma was classified as sarcoid-like, which might consist with tuberculosis, sarcoidosis and sarcoid-like reaction. Another 7 LNs with negative results were defined as false negatives, which were not confirmed by surgery. After multidisciplinary discussions held by clinicians from the thoracic surgery, radiology, respiratory oncology, and pathology departments in the hospital, the LNs were strongly suspected to be malignant and treatment was changed; hence, we cannot define them as negative.

**Table 2:** EBUS-TBNA performance of the suspect LNs

Sensitivity	94.02%
Specificity	100%
NPV	87.93%
PPV	100%
Final diagnosis	
Malignant	
*Other malignancies*	35
Colon and rectal adenocarcinoma	5
Esophageal cancer	3
SCC^†^ of head and neck	5
Cervical carcinoma	5
Breast cancer	4
Prostatic cancer	2
Liver cancer	4
Renal cancer	3
Malignant melanoma	2
Hemangiopericytoma	1
Thymic SCC^†^	1
*Lung cancer*	75
AC^‡^	30
SCC^†^	18
SCLC^§^	12
NSCLC^※^	10
Others	5
*Benign*	51
Sarcoid-like	11
Surveillance^¤^	27
Surgery	13

^†^SCC: Squamous cell carcinoma

^‡^AC: Adenocarcinoma

^§^SCLC: Small cell lung cancer

^※^NSCLC: Non small cell lung cancer

^¤^Surveillance: No progress after at least 6 months follow-up without changing of treatment

Overall, the sensitivity, specificity, NPV, and PPV were 94.02%, 100%, 87.93%, and 100%, respectively (Table 2).

The ultrasonographic features used were according to previous definitions as follows [13]:

1. Absence of a CHS in the LN: a missing flat, central, echogenic structure in the LN.
2. Short axis > 10 mm: presence of a small axis length greater than 10 mm.
3. Well-defined margins: > 50% echogenic line delimiting the LN.
4. Presence of central necrosis: presence of a central hypoechoic structure in the LN.
5. Round shape: the ratio of the long and small axis being less than 1.5.

Pearson’s Chi-square analysis was used to assess categorical variables. Univariate binary logistic regression was completed for each ultrasonographic feature. Among the features, CHS, margin and short axis were significantly predictive of malignancy (*p* < 0.05). Multivariate analysis revealed CHS and short axis to be independent factors of malignancy (Tables 3, 4, and 5).

**Table 3:** EBUS features presence in malignant and benign lymph nodes

Ultrasonographic feature	Malignant	Benign	Pearson X^2^ value	*p* value
Central hilar structure				
Presence	9 (45.0)	11 (55.0)	6.52	0.011*
Absence	108 (73.0)	40 (27.0)		
Short axis				
< 10 mm	15 (48.4)	16 (51.6)	8.12	0.004*
≥ 10 mm	102 (74.5)	35 (25.5)		
Margin				
Ill-define	98 (67.1)	48 (32.9)	3.35	0.067
Well-define	19 (86.4)	3 (13.6)		
Central necrosis				
Absence	112 (69.6)	49 (30.4)	2.16	0.323
Presence	5 (100.0)	0 (0.0)		
Round shape (long axis/short axis)				
≥ 1.5	29 (38.3)	29 (61.7)		
< 1.5	88 (72.7)	33 (27.3)		0.163

*: p < 0.05 defined as of statistical significance

**Table 4:** Univariate analyse for ultrasonographic features with logistic regression

Ultrasonographic feature	OR	SE	Z score	*p* value	95% CI
Central hilar structure	3.3	1.60	2.46	0.014*	1.27–8.56
Short axis	3.1	1.27	2.77	0.006*	1.39–6.93
Margin	3.1	2.00	1.75	0.080	0.87–11.00
Round shape	0.6	0.22	−1.39	0.165	0.30–1.23

*: p < 0.05 defined as of statistical significance

**Table 5:** Multivariate analyse for ultrasonographic features with logistic regression

Ultrasonographic feature	OR	SE	Z score	*p* value	95% CI
Central hilar structure	2.83	1.49	1.97	0.049*	1.01–7.95
Short axis	2.24	0.66	2.75	0.006*	1.26–3.98
Margin	3.00	2.03	1.63	0.103	0.80–11.27

*: p < 0.05 defined as of statistical significance

## Discussion

It is well established that the status of thoracic LNs has an important impact on treatment decision for patients with lung cancer. EBUS-TBNA is recommended as an effective and minimally invasive tool in the mediastinal and hilar assessment during initial diagnosis. However, other tumors and benign diseases also cause enlargement of the thoracic LNs. Especially after anti-tumor systemic treatment, it is difficult to determine the cause of the LN changes, the result of metastasis of primary tumors, a different lung cancer, or benign diseases such as tuberculosis and sarcoidosis. There are also reports on lung cancers developing resistance to molecular-targeted therapeutic agents through the expression of resistant mutations or even histological transformations after initial treatment [18, 19]. Clarifying the etiologies of lymphadenopathy is crucial to the choice of treatment. With the application of immunotherapy, some scholars observed sarcoid-like granulomatosis of the mediastinal LN as an immune response [1, 20], even suggesting an improved overall survival [1]. In our series, a patient suffered multiple mediastinal LN enlargements after immunotherapy. After EBUS-TBNA, cytology revealed a response to therapy. These findings illustrate the importance of obtaining a tissue diagnosis when imaging reveals enlarging LNs after anti-tumor treatments, as the differential diagnosis includes benign entities, such as sarcoid-like reactions, in addition to disease progression. Endobronchial ultrasound-guided sampling can help rule out malignant lymphadenopathy and minimize interruptions in treatment. However, in our series the most commonly occurring reason for LN enlargement was malignancy. The majority were LNs of lung cancer metastasis (75 LNs) and other malignancies (35 LNs) including colon and rectal adenocarcinoma, esophageal cancer, SCC of head and neck, cervical carcinoma, breast cancer, prostatic cancer, liver cancer, renal cancer, malignant melanoma, hemangiopericytoma, and thymic SCC.

The diagnostic value of EBUS for these LNs after treatment is not well demonstrated. In this study, the data of 168 LNs from 138 patients who underwent EBUS-TBNA were collected, and the sensitivity, specificity, NPV, and PPV were 94.02%, 100%, 87.93%, and 100%, respectively. The negative results were confirmed by surgery or more than 6 months of follow-up. If no further progress (enlargement) was observed during the 6-month follow-up without changing treatment regimen, it was confirmed to be truly negative. For non-tumor patients, a longer follow-up may be a better choice; however, for patients with definite tumors, a longer follow-up interval might be influenced or misled by many other factors, resulting in a confusing analysis. Overall, for the follow-up duration, we chose 6 months in accordance with other clinicians [21]. EBUS-TBNA in patients with previously treated lung cancer has been reported as effective, although the number of patients was limited [18, 22, 23]. Our result confirmed the high diagnostic yield of EBUS-TBNA for LNs post treatment, which is even higher than for LNs without any anti-tumor treatment.

Our series had a larger volume and included patients with lung cancer and other diseases, thus confirming a wider applicability of EBUS-TBNA. The procedures in our study were mainly performed in outpatient units; patients left the endoscopy center two hours after the procedure. No major or minor complications were observed in our series. This also proved the safety and practical value of EBUS-TBNA.

EBUS is not only concerned with the acquisition of specimens. Diagnostic value of endobronchial ultrasonographic features as a supplement to pathology diagnosis has attracted increasing attention in recent years [12, 15, 24]. EBUS-TBNA aims to gain enough sample to identify potential malignancy; meanwhile, the image acquired using EBUS-TBNA provides clinical information. There are often multiple LNs within the same nodal station, and EBUS may aid in determining the most suspicious LN, thus guiding the selection of LNs for TBNA during the process. In this way, EBUS features are thought to help improve the efficiency of biopsy. In addition, for a negative result or insufficient sample, ultrasonographic features have a complementary diagnostic value [25]. Through this procedure, clinicians may obtain valuable information that will aid in deciding how to proceed, which may mean further investigation including a repeated EBUS-TBNA or attempt to gain more tissue, or wait-and-observe. Many studies have explored various ultrasonographic features to find the optimal predictive model for noninvasive diagnosis of LNs using EBUS; for example, the Canada Lymph Node Score for prediction of malignancy in mediastinal LNs during endobronchial ultrasound. In a variety of multimodal analytic methods, as previously reported, EBUS ultrasonographic features, including CHS, margin, short axis, shape (round shape), coagulation necrosis sign/central necrosis, have relatively accurate predictive values for malignancy [12, 13, 15, 24]. Moreover, these factors are the most commonly conducted ones, with the gray scale mode. Other potential tools include elastography features and blood flow/vascularity pattern [12, 15]. Several scores or grade classifications including the Canada Lymph Node Score [13], vascular image patterns grade [16], and elastography grading [14, 26] were proposed, and real-world validation confirmed the usefulness [25].

After anti-tumor treatment, the LN may change, thus affecting ultrasonographic features. The predictive value of ultrasonographic features is not well explored. During our study, because of another study, we recorded the ultrasonographic features during EBUS process; hence, we collected and documented these data. In this retrospective study, the records of these image features were almost immediately obtained. In clinical settings, the simpler the features are, the better. Hence, we chose the simple ones with high interobserver-agreement, which could be obtained from gray mode. For these characteristics, we carried out univariate and multivariate analyses. The round shape is defined as long axis/short axis ratio of less than 1.5 [12]. In the univariate analysis, CHS, margin and short axis showed significant statistical difference (*p* < 0.05). In multivariate analysis, CHS and short axis showed statistically significant difference.

On this basis, EBUS images still have certain predictive value for LNs after treatment. Independent factors related to malignancy are CHS and short axis. Compared with previous reports about image characteristics of LNs, it seems that image divergence between benign and malignant LNs is smaller after treatment. So for suspicious LNs after treatment we should rely more on sampling. Without doubt, the pathological results of specimens for untreated LNs are irreplaceable in determining the nature of LNs.

For negative EBUS-sampling pathologies, if EBUS features could be taken into account, interruption or delay in treatment caused by repeated investigations might be avoided. Specimens with a negative pathologic result obtained by EBUS-TBNA were likely to be true-negative findings as reported by many scholars [27–29], and the EBUS-false-negative had a favorable survival that was similar to that of true-negative patients [30]. In our case, besides the surgery patients, four of the EBUS-negative(sonographic negative) patients had repeated EBUS-TBNA and remained negative. After at least 6 months, there was no progress observed in radiologic findings. Considering NPV of EBUS-TBNA sampling, in patients with negative pathology and low-risk image features, instead of ceasing or changing therapeutic regimen, more conservative approaches might be considered even after previous therapy for the tumor. In contrast, all the sonographic findings of all 7 false negative cases contained 2 or 3 suspicious features for malignancy.

As a minimally invasive procedure, EBUS-TBNA could be conducted in outpatient settings. As per our study, considering its efficacy, EBUS-TBNA could be applied more aggressively after or during anti-tumor therapy. Thus, by helping rule out malignant lymphadenopathy and minimize interruptions in treatment, or quickly discover resistance to initial treatment, EBUS-TBNA could be more widely applied to improve patient outcomes.

The present study had some limitations. It was a single institution analysis and the results were based on a small number of samples. In clinical practice, some questions are not clearly confirmed. For example, the false negative-cases (7 lymph nodes of 6 patients) were not confirmed by surgery or other procedures thus no positive specimen was obtained. Multi-center prospective trials will be needed to support the efficacy of EBUS-TBNA for the repeated evaluation of recurrence as well as treatment effectiveness during the course of cancer treatment.

## Conclusion

EBUS-TBNA for patients with previously treated cancer showed a high diagnostic yield and was found to be safe. With the increasing need of tissue for managing optimal treatment, EBUS-TBNA after or during the course of treatment could play a more active role. Ultrasonographic features like CHS and short axis are potential independent factors related to malignant lymphadenopathy. Recognizing specific imaging features could help clinicians in decision-making especially under negative TBNA situations. Larger prospective investigations are needed to confirm the NPV of EBUS-TBNA after treatment for malignancies.

## Data Availability

The data that support the findings of this study are available from Peking University Cancer Hospital & Institute but restrictions apply to the availability of these data, which were used under license for the current study, and so are not publicly available. Data are however available from the corresponding author (Professor Qi Wu) upon reasonable request and with permission of Peking University Cancer Hospital & Institute.

## Abbreviations

EBUS-TBNA: Endobronchial ultrasound-guided transbronchial needle aspiration
CHS: Central hilar structure
CT: Computed tomography
PET: Positron emission tomography
LN: Lymph node
NPV: Negative predictive value
PPV: Positive predictive value
SCC: Squamous cell carcinoma

## References

[1] Cabanie C, Ammari S, Hans S, Pobel C, Laparra A, Danlos FX (2021). Outcomes of patients with cancer and sarcoid-like granulomatosis associated with immune checkpoint inhibitors: A case-control study. Eur J Cancer.

[2] Luo GY, Cai PQ, He JH, Li JJ, Li Y, Shan HB (2013). Application of endobronchial ultrasound-guided transbronchial needle aspiration in the management of mediastinal and hilar lymphadenopathy without intrapulmonary mass: experience from the largest cancer center of southern China. Cell Biochem Biophys.

[3] Oezkan F, Eisenmann S, Darwiche K, Gassa A, Carbone DP, Merritt RE (2021). Linear endobronchial ultrasound in the era of personalized lung cancer diagnostics—a technical review. J Clin Med.

[4] Sakakibara R, Inamura K, Tambo Y, Ninomiya H, Kitazono S, Yanagitani N (2017). EBUS-TBNA as a promising method for the evaluation of tumor PD-L1 expression in lung cancer. Clin Lung Cancer.

[5] Oezkan F, Herold T, Darwiche K, Eberhardt WEE, Worm K, Christoph DC (2018). Rapid and highly sensitive detection of therapeutically relevant oncogenic driver mutations in EBUS-TBNA specimens from patients with lung adenocarcinoma. Clin Lung Cancer.

[6] Stoy SP, Segal JP, Mueller J, Furtado LV, Vokes EE, Patel JD (2018). Feasibility of endobronchial ultrasound-guided transbronchial needle aspiration cytology specimens for next generation sequencing in non-small-cell lung cancer. Clin Lung Cancer.

[7] Giuliani ME, Hope A, Mangona V, Guckenberger M, Mantel F, Peulen H (2017). Predictors and patterns of regional recurrence following lung SBRT: a report from the Elekta Lung Research Group. Clin Lung Cancer.

[8] Zhao JJ, Chan HP, Soon YY, Huang Y, Soo RA, Kee ACL (2022). A systematic review and meta-analysis of the adequacy of endobronchial ultrasound transbronchial needle aspiration for next-generation sequencing in patients with non-small cell lung cancer. Lung Cancer.

[9] Tone M, Inomata M, Awano N, Kuse N, Takada K, Minami J (2021). Comparison of adequacy between transbronchial lung cryobiopsy samples and endobronchial ultrasound-guided transbronchial needle aspiration samples for next-generation sequencing analysis. Thorac Cancer.

[10] Yang H, Hall SRR, Yao F (2020). The value of PD-L1 expression in metastatic lymph nodes of advanced non-small cell lung cancer. Chest.

[11] Leong TL, Christie M, Kranz S, Pham K, Hsu A, Irving LB (2017). Evaluating the genomic yield of a single endobronchial ultrasound-guided transbronchial needle aspiration in lung cancer: meeting the challenge of doing more with less. Clin Lung Cancer.

[12] Zhi X, Chen J, Xie F, Sun J, Herth FJF (2021). Diagnostic value of endobronchial ultrasound image features: a specialized review. Endosc Ultrasound.

[13] Hylton DA, Turner S, Kidane B, Spicer J, Xie F, Farrokhyar F (2020). The Canada Lymph Node Score for prediction of malignancy in mediastinal lymph nodes during endobronchial ultrasound. J Thorac Cardiovasc Surg.

[14] Sun J, Zheng X, Mao X, Wang L, Xiong H, Herth FJF (2017). Endobronchial ultrasound elastography for evaluation of intrathoracic lymph nodes: a pilot study. Respiration.

[15] Wang L, Wu W, Hu Y, Teng J, Zhong R, Han B (2015). Sonographic features of endobronchial ultrasonography predict intrathoracic lymph node metastasis in lung cancer patients. Ann Thorac Surg.

[16] Nakajima T, Anayama T, Shingyoji M, Kimura H, Yoshino I, Yasufuku K (2012). Vascular image patterns of lymph nodes for the prediction of metastatic disease during EBUS-TBNA for mediastinal staging of lung cancer. J Thorac Oncol.

[17] Nosotti M, Ferrari M, Righi I, Mendogni P, Damarco F, Cattaneo M (2021). The role of sonographic patterns during endobronchial ultrasound-transbronchial needle aspiration for lung cancer staging: a narrative review. Mediastinum.

[18] Fujiwara T, Nakajima T, Inage T, Sata Y, Yamamoto T, Sakairi Y (2021). Endobronchial ultrasound-guided transbronchial needle aspiration in patients with previously treated lung cancer. Surg Today.

[19] Guarize J, Casiraghi M, Donghi S, Casadio C, Diotti C, Filippi N (2017). EBUS-TBNA in PET-positive lymphadenopathies in treated cancer patients. ERJ Open Res..

[20] Frohlich M, Wang H, Sakr L (2020). Sarcoid-like reaction discovered on EBUS-TBNA of intrathoracic lymph nodes during immunotherapy for metastatic melanoma. J Immunother.

[21] Chi J, Lian SS, Yang Q, Luo GY, Xu GL (2020). The utility of EBUS-TBNA in the diagnosis of suspected intrathoracic recurrence after esophageal cancer surgery. Jpn J Clin Oncol.

[22] Anraku M, Pierre AF, Nakajima T, de Perrot M, Darling GE, Waddell TK (2011). Endobronchial ultrasound-guided transbronchial needle aspiration in the management of previously treated lung cancer. Ann Thorac Surg.

[23] Kim J, Kang HJ, Moon SH, Lee JM, Kim HY, Lee GK (2019). Endobronchial ultrasound-guided transbronchial needle aspiration for re-biopsy in previously treated lung cancer. Cancer Res Treat.

[24] Fujiwara T, Yasufuku K, Nakajima T, Chiyo M, Yoshida S, Suzuki M (2010). The utility of sonographic features during endobronchial ultrasound-guided transbronchial needle aspiration for lymph node staging in patients with lung cancer: a standard endobronchial ultrasound image classification system. Chest.

[25] He RX, Hylton DA, Bedard EL, Johnson S, Laing B, Valji A (2022). Clinical validation of the Canada lymph node score for endobronchial ultrasound. Ann Thorac Surg.

[26] Izumo T, Sasada S, Chavez C, Matsumoto Y, Tsuchida T (2014). Endobronchial ultrasound elastography in the diagnosis of mediastinal and hilar lymph nodes. Jpn J Clin Oncol.

[27] Mehta HJ, Tanner NT, Silvestri G, Simkovich SM, Shamblin C, Shaftman SR (2015). Outcome of patients with negative and unsatisfactory cytologic specimens obtained by endobronchial ultrasound-guided transbronchial fine-needle aspiration of mediastinal lymph nodes. Cancer Cytopathol.

[28] Taverner J, Cheang MY, Antippa P, See K, Irving LB, Steinfort DP (2016). Negative EBUS-TBNA predicts very low prevalence of mediastinal disease in staging of non-small cell lung cancer. J Bronchology Interv Pulmonol.

[29] Hylton DA, Kidane B, Spicer J, Turner S, Churchill I, Sullivan K (2021). Endobronchial ultrasound staging of operable non-small cell lung cancer: do triple-normal lymph nodes require routine biopsy?. Chest.

[30] Hwangbo B, Park EY, Yang B, Lee GK, Kim TS, Kim HY (2021). Long-term survival according to N stage diagnosed by endobronchial ultrasound-guided transbronchial needle aspiration in non-small cell lung cancer. Chest.

